# Yeast and Filamentous Fungi Microbial Communities in Organic Red Grape Juice: Effect of Vintage, Maturity Stage, SO_2_, and Bioprotection

**DOI:** 10.3389/fmicb.2021.748416

**Published:** 2021-12-24

**Authors:** Sara Windholtz, Emmanuel Vinsonneau, Laura Farris, Cécile Thibon, Isabelle Masneuf-Pomarède

**Affiliations:** ^1^Univ. Bordeaux, INRAE, Bordeaux INP, UR OENOLOGIE, EA 4577, USC 1366, ISVV, Villenave d’Ornon, France; ^2^Institut Français de la Vigne et du Vin, Blanquefort, France; ^3^Bordeaux Sciences Agro, Gradignan, France

**Keywords:** vinification without SO_2_, bioprotection, biodiversity, prefermentary stages, maturity level

## Abstract

Changes are currently being made to winemaking processes to reduce chemical inputs [particularly sulfur dioxide (SO_2_)] and adapt to consumer demand. In this study, yeast growth and fungal diversity were investigated in merlot during the prefermentary stages of a winemaking process without addition of SO_2_. Different factors were considered, in a two-year study: vintage, maturity level and bioprotection by the adding yeast as an alternative to SO_2_. The population of the target species was monitored by quantitative-PCR, and yeast and filamentous fungi diversity was determined by 18S rDNA metabarcoding. A gradual decrease of the α-diversity during the maceration process was highlighted. Maturity level played a significant role in yeast and fungal abundance, which was lower at advanced maturity, while vintage had a strong impact on *Hanseniaspora* spp. population level and abundance. The presence of SO_2_ altered the abundance of yeast and filamentous fungi, but not their nature. The absence of sulfiting led to an unexpected reduction in diversity compared to the presence of SO_2_, which might result from the occupation of the niche by certain dominant species, namely *Hanseniaspora* spp. Inoculation of the grape juice with non-*Saccharomyces* yeast resulted in a decrease in the abundance of filamentous fungi generally associated with a decline in grape must quality. Lower abundance and niche occupation by bioprotection agents were observed at the overripened stage, thus suggesting that doses applied should be reconsidered at advanced maturity. Our study confirmed the bioprotective role of *Metschnikowia pulcherrima* and *Torulaspora delbrueckii* in a context of vinification without sulfites.

## Introduction

Grape must contains a number of microorganisms: yeast, bacteria and filamentous fungi (Barata et al., 2012). Various parameters have been shown to impact grape must microbial diversity, such as the farming system (Comitini and Ciani, 2008; Martins et al., 2014; Grangeteau et al., 2017; Agarbati et al., 2019), vintage (Grangeteau et al., 2017), geographical area (Bokulich et al., 2014), cultivar (Fleet, 2008; Zhang et al., 2019), ripening stage (Martins et al., 2012, 2014), sanitary status of the grape berries (Barata et al., 2012), prefermentary operations (Epifanio et al., 1999; Grangeteau et al., 2017), or the use of SO_2_ (Bokulich et al., 2015). Filamentous fungi most often found on grape berries and in must are generally undesirable: *Cladosporium* spp. causes rot, which reduces yield and affects the quality of wines (Briceño et al., 2009; Latorre et al., 2011), *Alternaria* spp., *Aspergillus* spp. and *Penicillium* spp. produce mycotoxins (Serra et al., 2003; Bui-Klimke and Wu, 2015; Rantsiou et al., 2020). Additionally, the association of *B. cinerea* with other molds like *Penicillium* spp. and *Rhizopus* spp. can lead to result in production of wine defects (Barata et al., 2012). Non-*Saccharomyces* yeasts, such as *Hanseniaspora*, *Starmerella*, *Pichia*, *Metschnikowia*, *Zygoascus*, *Issatchenkia* or *Rhodotorula* genus, are considered dominant in the prefermentary stages (Fleet, 2008; Zott et al., 2010; Barata et al., 2012; Albertin et al., 2014). Some non-*Saccharomyces* species/strains are considered detrimental for wine quality, others represent resources to promote innovation in the oenological sector (Padilla et al., 2016; Berbegal et al., 2017; Tufariello et al., 2021).

Sulfur dioxide (SO_2_) has been used in enology since the 1800s (Pasteur, 1866; Ladrey, 1871) for its antimicrobial, antioxidant (Waterhouse et al., 2016) and antioxydasic (Ough and Crowell, 1987; Son et al., 2001) properties, throughout the winemaking process, from vatting to bottling. During the winemaking process, free SO_2_ has been reported to neutralize and eliminate spoilage microorganisms, delay growth of lactic acid bacteria, and limit growth of non-*Saccharomyces* yeast (Henick-Kling et al., 1998; Takahashi et al., 2014). A negative effect of SO_2_ has been demonstrated on maximum populations of *Hanseniaspora*, followed by a faster decline during fermentation (Andorrà et al., 2008; Albertin et al., 2014; Takahashi et al., 2014). Sulfur dioxide also facilitates implantation of the sulfite-tolerant *Saccharomyces cerevisiae* species (Constantì et al., 1998; Henick-Kling et al., 1998; Albertin et al., 2014), in particular due to the presence of sulfite pumps (Park and Bakalinsky, 2004; Zimmer et al., 2014; Marullo et al., 2020).

Nowadays, the use of preservatives is a source of controversy. Use of SO_2_ in producing wines is pointed to as a cause of intolerance (Warner et al., 2000; Timbo et al., 2004; Vally et al., 2009). In addition, consumer views of the agrifood products they ingest have changed, as they have become more aware of preservative compounds and thus wish to eat and drink more “healthily” (D’Amico et al., 2016; Apaolaza et al., 2017). Consumers are willing to pay more for so-called “sustainable” wines, which they consider more environmentally friendly, such as sulfite-free wines (Poveda et al., 2005; Forbes et al., 2009; Pérès et al., 2018; Galati et al., 2019). Furthermore, faced with global warming, as wines become richer with higher pH levels, the effectiveness of SO_2_ will be reduced, with significant consequences for fermentation and the management of microbial populations (De Orduna, 2010; Drappier et al., 2019).

Faced with these issues, winemakers have had to reduce the use of sulfur dioxide in their wine production, as wine is the main food source of sulfites in terms of doses. Different alternatives are available on the market, such as adjuvants like chitosan and dimethyl decarbonate, or physical methods such as filtration or heat treatments (Lisanti et al., 2019). Recently, bioprotection by adding yeasts has been proposed as a new alternative. This involves adding antagonist cultures (or their metabolic products) to inhibit pathogens and/or extend shelf life, while minimizing the impact on the sensory properties of the treated product (Lücke, 2000). Simonin et al. (2018) showed the effectiveness of *Torulaspora delbrueckii* in colonizing the medium in an aligoté must, a white Burgundy grape variety. In a preliminary study, the use of two species as bioprotection agents was tested in a context of Bordeaux region: *Torulaspora delbrueckii* and *Metschnikowia pulcherrima* in merlot must (Windholtz et al., 2021a). Their implantation was confirmed as limiting the abundance of filamentous fungi.

Furthermore, Johnson et al. (2020) showed the impact of different bioprotection products on growth of *Hanseniaspora* spp. during a cold prefermentary maceration.

Bioprotection by adding non-*Saccharomyces* yeasts would therefore seem to be a promising alternative to sulfur dioxide during the vinification process. The impact of the absence of sulfur dioxide and use of bioprotection yeasts on the grape juice microbial community should be evaluated in several contexts in terms of grape must microbial and chemical composition and of ripening stages, particularly advanced maturity in a context of global warming. Regardless of grape juice chemical composition, the maturity stage has been shown to impact the size and composition of the microbial communities on the grape-berry surface, with significantly higher population levels and diversity at the overripened stage (Martins et al., 2012, 2014). Through two complementary molecular biology approaches (Q-PCR and high throughput sequencing), this study aims to provide indications about the impact of the harvest date, absence of SO_2_ and bioprotection agents on yeast and filamentous fungi. The impact of the harvest date, absence of SO_2_ and use of a bioprotection alternative on yeast dynamics and diversity was studied in merlot during the prefermentary stages, in comparison with the traditional use of SO_2_, over two consecutive vintages. From the results of high throughput sequences for the filamentous fungi, intra- and inter-sample biodiversity indices were determined, thus allowing yeast and fungal community composition to be characterized.

## Materials and Methods

### Experimental Treatments

In 2017 and 2018, merlot N. grapes (*Vitis vinifera L.*) were harvested manually from a wine estate applying an organic farming system in the “Entre-deux-Mers” area. They were picked at two ripening stages: technological maturity as defined by the winemaker (maturity “I”) and advanced maturity after which harvest was carried out one week later (maturity “II”). Precipitation during the ripening period (August, September, and October) was 115 and 111 mm in 2017 and 2018, respectively. Average minimal temperatures were 11.5–16.6°C in 2017 and 7–17.1°C in 2018, and average maximal temperatures were 21.2–28°C for 2017 and 14.5–29.5°C in 2018. The 2017 vintage was characterized as an early vintage due to rains at the beginning of September (72 mm) causing an early start to the harvest (Geny et al., 2018), while 2018 had ideal conditions for the harvest (precipitation in September: 3 mm) with higher sugar content (Geny et al., 2019).

**FIGURE 1:**
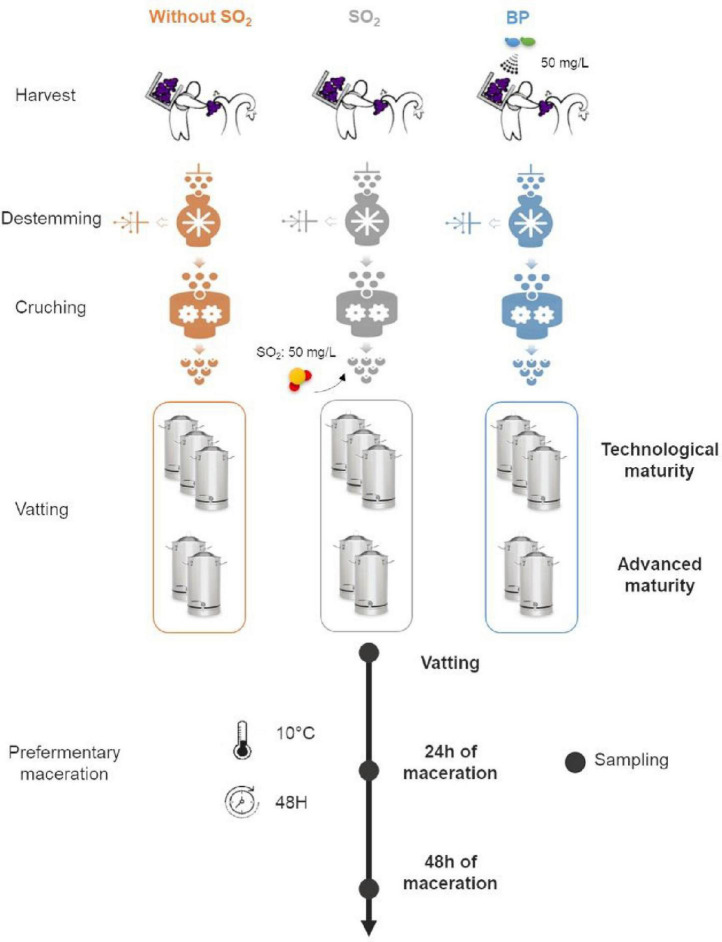
Experimental design of the study.

The same plot was harvested in 2017 and 2018, collecting the grapes from every other row for the two maturity stages. Three winemaking processes were followed: 50 mg/L of bioprotection [*Torulaspora delbrueckii* and *Metschnikowia pulcherrima* species (Zymaflore^®^ Egide – Laffort)] applied directly to the grapes following the manufacturer’s indications for rehydration and without addition of SO_2_ (“BP” modality), 50 mg/L of SO_2_ at vatting (“SO_2_” modality), and without any addition (“Without SO_2_” modality). Grapes were distributed evenly for each treatment and were then crushed and destemmed. Winemaking was conducted in tanks of 35L in triplicate (maturity I) and duplicate (maturity II) for each modality. Prefermentary maceration was carried out at 10°C before inoculation (200 mg/L) with commercial Active Dry Yeast (ADY) *Saccharomyces cerevisiae* after 48H. During prefermentary maceration, 10 mL of must was sampled in sterile conditions at three times (Vatting, 24H of maceration, 48H of maceration corresponding to “Stage” parameter) and transported immediately on ice to the laboratory for processing (Figure 1).

### DNA Extraction

Samples of 10 mL of must were centrifuged at 9,000 rpm for 10 min, and then pellets were rinsed twice with 2 mL 0.05 M EDTA pH8 and conserved at −20°C until subsequent DNA extraction. A FastPrep-24 instrument (MP Biomedicals, Illkirch, France) was used for DNA extraction: 200 μl of glass beads (acid-washed, Ø 0.1 mm, Sigma, Lyon, France) and 1 ml of EDTA 0.05 M were added to the frozen pellet. The protocol described by Zott et al. (2010) was followed until complete extraction. DNA was conserved at −20°C.

### Population Level of Target Microorganisms by Quantitative PCR

The quantitative PCR (Q-PCR) method was used to monitor population dynamics of *Torulaspora delbruecki* and *Hanseniaspora* spp. with primers described in Zott et al. (2010), and *Metschnikowia pulcherrima* with primers described by García et al. (2017) (Supplementary Table 1). For quantification, standard curves were built for each yeast species in triplicate, using 10-fold serial dilutions of fresh culture in pasteurized red must. On must samples, quantitative PCRs to monitor *Botrytis cinerea* spores were obtained using Bc3R and Bc3F primers (Diguta et al., 2010).

### Meta-Barcoding and High-Throughput Sequencing Analysis

FR1 and FF390 primers were used to target 18S rDNA (David et al., 2014). First, the 18S rDNA gene fragment was amplified with an **adapter** for Amplicon PCR Reverse Primer (5′**GTCTCGTGGGCTCGGAGATGTGTATAAGAGACA** + FR1) and for Amplicon PCR Forward (5′**TCGTCGGCA GCGTCAGATGTGTATAAGAGACAGCG** + FF390). This PCR was taken in the laboratory: reactions were cycled for 3 min at 95°C, then for 35 cycles of 98°C for 30 s, 52°C for 30 s and 72°C for 60 s, then followed by a final extension period of 8 min at 72°C. The mix PCR was composed of 2.5 μL dilute template (DNAs standardized to 5 ng/μL), 5 μL each Amplicon PCR Primer 1 μM, 12.5 μL 2X KAPA HiFi HotStart Ready Mix (Roche, Bâle, Suisse). After the first amplification, a second PCR was carried out by the Plateforme Genome-Transcriptome of Bordeaux added indices and Illumina sequencing adapters with the Nextera^®^XT Index Kit. For the Illumina paired-end library, normalized pool libraries were prepared and clusters generated, and 2*250 bp paired-end sequencing (MiSeq Kit NANO v2) was performed on an Illumina MiSeq instrument. A total of 90 samples (3 modalities at 3 stages in triplicate for maturity I and 3 modalities at 3 stages in duplicate for maturity II, during two vintages) were sequenced by Illumina Miseq.

### Sequence Analysis

Sequence cleaning was carried out on the FROGS (Find Rapidly OTUs with Galaxy Solution) pipeline (Escudié et al., 2017) with the pre-process steps (pared end assembled with 5′ primer and 3′ primer, with expected length (<300 and >400 bps) and without N), SWARM clustering (Mahé et al., 2015), removal of chimera with VSEARCH (Rognes et al., 2016), and removal of singletons and contaminants. The Silvia132 18S database (Quast et al., 2012) was used as the database for the taxonomic assignment of OTUs (Operational Taxonomic Units). Sequences with <97% identity and <95% of coverage were deleted by filtration on BLAST. An affiliation postprocess was used to resolve inclusive amplicon ambiguities and aggregate OTUs based on alignment metrics. Finally, OTUs corresponding to *Vitis* sp. were removed.

### Statistical Analysis

The Phyloseq package (McMurdie and Holmes, 2013) was used on the Rstudio software (RStudio Team, 2020) to calculate α-diversity [Shannon index, inv-Simpson index and the species richness estimator (chao1)]. The ANOVA test and T-test were used to determine the impact of different parameters on α-diversity after data was assumed to be normally distributed (Shapiro-Wilks normality test, *p* > 0.05) and variance homogeneity was verified (Leven test, *p* > 0.05). Then, the Tukey *post hoc* test (*p* < 0.05) was used to find significant differences between the modalities (represented by different letters). The Ggplot2 package (Wickham et al., 2016, 2) was used for graphic representations.

From normalized sequences based on the sample which had the least sequences, β-diversity was determined with Jaccard (qualitative approach) and Bray-Curtis distances (quantitative approach). A principal coordinate analysis (PCoA) was performed to visualize the matrix obtained according to these two distances. A permutational multivariate analysis of variance (Manova) was applied with the Adonis function from the vegan package to determine the significant effect of the parameters on β-diversity (Oksanen et al., 2013).

## Results

Merlot grapes at two grape ripening stages (technological and advanced maturity) were processed applying three different prefermentary treatments, Bioprotection without SO_2_ (“BP” modality), 50 mg/L of SO_2_ at vatting (“SO_2_” modality), and without any addition (“Without SO_2_” modality), over two consecutive vintages. Then, different combinations in triplicate for maturity I and in duplicate for maturity II (due to the low harvest quantity) were considered for further analysis. Analysis of the must and quantification of *Botrytis cinerea* spores are presented in the Supplementary Table 2. Sugar content and pH were higher for maturity II than maturity I for both vintages (Supplementary Table 2). No significant differences were found between the two vintages regarding the quantification of the spores of *Botrytis cinerea*; however, on average, the quantity of spores was higher at technological maturity than at advanced maturity in both 2017 and 2018 (ANOVA, *p*-value = 0.003754^**^). In addition, the quantity of spores was significantly higher in the presence of SO_2_ than in the control without SO_2_ for both vintages (ANOVA, *p*-value = 0.03703*). Chemical analyses of the wines after alcoholic fermentation and total SO_2_ after malolactic fermentation are given in Supplementary Table 3. As expected, ethanol and pH were higher for advanced maturity and total SO_2_ was quantified only for the SO_2_ treatment; volatile acidity (0.09–0.14 g/L of acetic acid) was low for all experiments.

### Impact of Different Factors on Population Levels of *Hanseniaspora* spp. and Non-*Saccharomyces* Yeast Used as Bioprotection

**FIGURE 2:**
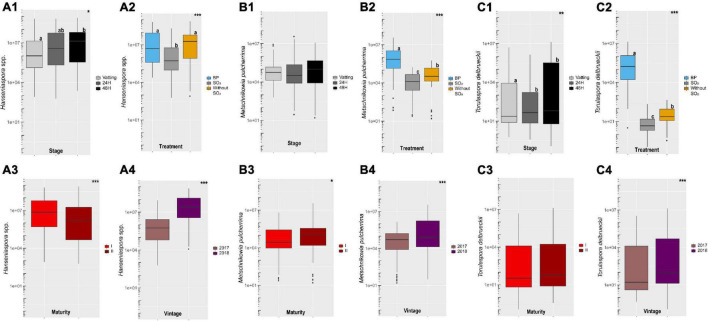
Boxplot of population levels obtained by Q-PCR (cells/mL) for *Hanseniaspora* spp. **(A)**, *Metschnikowia pulcherrima* **(B)**, *Torulaspora delbrueckii* **(C)** according to Stage (1) (Vatting, 24H and 48H of maceration), Treatment (2) (Bioprotection (BP), SO_2_ and Without SO_2_), Maturity (3) [technological (I) and advanced maturities (II)] and Vintage (4) (2017 and 2018) parameters. Significance is indicated as follow: * significant at 5%, ** significant at 1%, *** significant at 0.1% (ANOVA and T-test) and boxplot with different letters differ significantly (HSD-test).

Sampling was carried out at vatting, 24H and 48H of maceration at 10°C and population levels of *Hanseniaspora* spp. (A), *Metschnikowia pulcherrima* (B) and *Torulaspora delbrueckii* (C) were evaluated by Q-PCR (Supplementary Figure 1). Data are given in Figure 2 according to different parameters (stage, treatment, maturity and vintage). Population levels of *Hanseniaspora* spp. increased during prefermentary maceration (Figure 2A1). A strong vintage effect (Figure 2A4) was noted, with 25.8% of the variance explained (Supplementary Table 4). Population level was higher in 2018 (median: above 10^7^ cells/mL) than 2017 (median: below 10^6^ cells/mL). The treatment explained 8.6% of the total variance, with significantly lower population levels for the SO_2_ modality compared to the others. Use of bioprotection yeast did not limit growth of *Hanseniaspora* spp. (Figure 2A2) compared to SO_2_ addition. It is important to note a significant interaction for Vintage:Stage (10.5%) and Treatment:Stage (6.8%).

The population level of *Metschnikowia pulcherrima* was stable during cold maceration (Figure 2B1), unlike *Torulaspora delbrueckii*, which increased significantly (Figure 2C1). The indigenous population of *Metschnikowia pulcherrima* (median: above 10^4^ cells/L, Figure 2B2) was high compared to *Torulaspora delbrueckii* (median: below 10^2^ cells/L, Figure 2C2) and both species were impacted negatively by SO_2_ addition (Figure 2B2 and Figure 2C2). As expected, population levels of both non-*Saccharomyces* yeast in the bioprotection modality were significantly higher, due to the inoculation at 50 mg/L, except for *Metschnikowia pulcherrima* in 2017, maturity II (Supplementary Figure 1).

The growth of *Torulaspora delbrueckii* during prefermentary maceration and its low indigenous population level resulted in a higher percentage of variance in population levels explained by the treatment (77.4%) than for *Metschnikowia pulcherrima* (29.7%).

### Impact of the Different Parameters on Yeast and Filamentous Fungi Diversity

To evaluate yeast and fungal microbial diversity during prefermentary stages, the 18S rDNA gene was analyzed by high-throughput sequencing (HTS). 1,134,820 sequences were obtained, i.e., 12,609 sequences per sample. 1,042,017 sequences were retained after a pre-process step containing paired-end assembled, with 5′ and with 3′ primers, with expected length (between 300 and 400 bps) and without ambiguous characters (N) in their sequences. The SWARM clustering step generated 48,968 OTUs. After cleaning of chimera (−11,222 OTUs and −118,732 sequences), singletons (−36,190 OTUs, 36,785 sequences) and the OTU affiliations, 1,554 OTUs and 886,481 sequences were retained. A filtration on BLAST with the identity (>97%) and coverage (>95%) parameters was used and *Vitis* sp. OTUs were deleted from the dataset (−1374 OTUs and −51,377 sequences). Finally, an aggregation of OTUs based on alignment metrics was performed to obtain 180 OTUs and 835,104 sequences, i.e., 9,279 sequences per sample.

*Ascomycota* phylum, the mostly widely represented in the OTU dataset (99.46%), was made up of 4 major Classes: *Saccharomycetes* at 72.28% (represented by *Hanseniaspora* (58.9%), *Saccharomyces* (19.79%); *Torulaspora* (14.91%) and *Metschnikowia* (5.7%) genus), *Dothideomycetes* at 13.70% (represented by *Aureobasidium* (67%) and *Cladosporium* (29.3%) genus mostly), *Eurotiomycetes* at 8.66% [represented by *Aspergillus* genus (98.9%)] and *Leotiomycetes* at 4.84% represented by *Botrytis* genus (95.8%).

**FIGURE 3:**
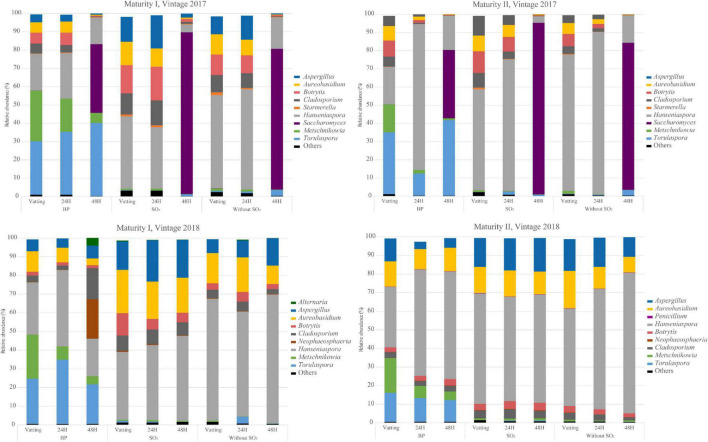
Relative abundances (%) of 9 major genus of *Ascomycota* phylum for all sample during prefermentary stages (Vatting, 24H and 48H of maceration) in two vintages (2017 and 2018) and two maturities [technological (I) and advanced maturities (II)]. Bioprotection (BP), SO_2_ and Without SO_2_ treatments in early stages of winemaking. Results of each sample is the mean of biological replicats (*n* = 3 technological maturity, *n* = 2 advanced maturity).

The relative abundance obtained for each experiment is presented in Figure 3 and Supplementary Figure 2. Overall, filamentous fungi abundance gradually decreased during the prefermentary stages. The diversity and abundance of OTUs differed from one vintage to another. *Alternaria* and *Neophaerosphaeria* were identified only in 2018, with an unexpected high abundance for maturity I after 48H. The relative abundance of *Aureobasidium* was higher in 2018 than in 2017, and *Aspergillus* was identified systematically in 2018 but not in 2017 (maturity II). The relative abundance of *Botrytis cinerea* spores showed a greater presence of *Botrytis cinerea* at technological maturity than at advanced maturity, according to the Q-PCR analysis and lower abundance at maturity I at vatting and 24H for bioprotection for both vintages. A spontaneous population of *Saccharomyces* colonized the grape juice at the end of maceration in 2017, but was absent in 2018. *Hanseniaspora* was the dominant OTU whatever the modality, stage and vintage considered. Both bioprotection species were present at the vatting stage for all the trials, with a higher abundance of *Torulaspora delbrueckii* than *Metschnikowia pulcherrima* during prefermentary maceration. The abundance of OTUs related to filamentous fungi (*Aspergillus*, *Cladosporium* and *Botrytis)* was lower when bioprotection yeasts was applied (Supplementary Figure 2), as was *Saccharomyces* abundance at the end of maceration in 2017. Between the two maturity levels, both vintages combined, the relative abundance of both non-S*accharomyces* species were lower at maturity II and the abundance of *Hanseniaspora* was higher at advanced maturity.

**FIGURE 4:**
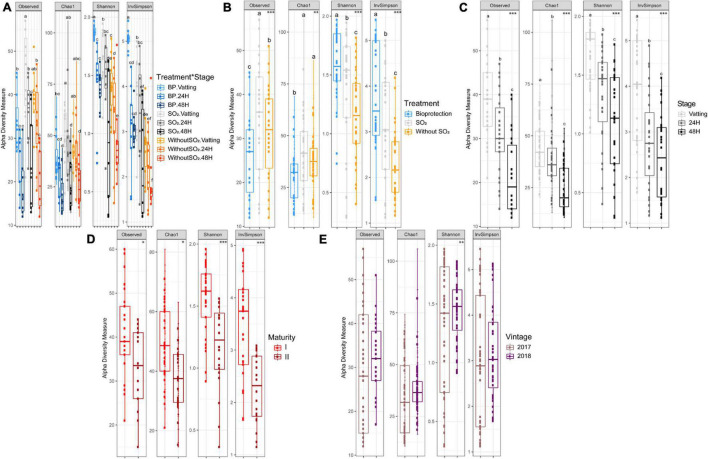
α – diversity by four indices (Observed, Chao1, Shannon, InvSimpson) according to Treatment^∗^Stage **(A)**, Treatment **(B)** (Bioprotection (BP), SO_2_ and Without SO_2_), Stage **(C)** (Vatting, 24H and 48H of maceration), Maturity **(D)** [technological (I) and advanced maturities (II)] and Vintage **(E)** (2017 and 2018). Significance is indicated as follows: * significant at 5%, ** significant at 1%, *** significant at 0.1% (ANOVA and T-test), and boxplot with different letters differ significantly (HSD-test).

In order to characterize the interspecific biodiversity, different indices were calculated: Observed and Chao1 providing qualitative information only, then the Shannon and InvSimpson indices, which take into account both the abundance and nature of the OTUs (Figure 4). By combining all the data (Figure 4A), significant differences were obtained only for InvSimpson. Indeed, BP – Vatting had a significantly higher index, and the Without SO_2_ – 48H modality was significantly the lowest.

Considering the Treatment parameter (Figure 4B), significant differences could be observed for all indices: the number of OTUs was lower with bioprotection yeasts, but due to the significant presence of *Torulaspora delbrueckii* and *Metschnikowia pulcherrima*, its Shannon and InvSimpson index was significantly higher. Interestingly, the absence of sulfiting led to a decrease in both the Shannon and InvSimpson indices.

The Stage parameter affected all the indices that were calculated (Figure 4C): biodiversity of the yeast and fungal microbial community decreased gradually during the maceration process. Concerning maturity (Figure 4C), the Shannon and InvSimpson indices were significantly lower with advanced maturity (II), as were the observed α-diversity and Chao index, but to a lesser extent (*p*-value = 0.05). Finally, the Vintage parameter did not have a significant impact on α-diversity, except for the Shannon index, which was lower for the 2017 vintage (Figure 4E).

The proportion of variance explained by each parameter and their interactions is presented in Table 1. The Stage parameter explained the largest part of variance for the four indices, followed by Treatment. Maturity accounted for variance of the quantitative indices (Shannon: 11.9% and InvSimpson: 13.6%). The Vintage*Stage interaction was significant and had an impact on the qualitative biodiversity indices, and to a lesser extent, on the Shannon index. The proportion of variance not explained by the parameters considered here (Residuals) was relatively low, except for the Chao1 index for which it was 21%.

**TABLE 1:** Percentage of variance explained by Treatment (Without SO_2_, SO_2_ and Bioprotection), Stage, Vintage, Maturity factors and these interactions for different α biodiversity indices.

	Observed		Chao1		Shannon		InvSimpson	
Treatment	10.1%	***	8.5%	***	15.7%	***	25.8%	***
Vintage	1.8%	**	2.3%	**	3.7%	***	0.8%	**
Stage	41.2%	***	33.3%	***	28.6%	***	24.8%	***
Maturity	2.9%	***	4.2%	**	11.9%	***	13.6%	***
Treatment:Vintage	0.4%		0.2%		1.1%	*	1.0%	*
Treatment:Stage	1.4%		1.5%		4%	***	7.7%	***
Treatment: Maturity	2.8%	**	3.5%	*	1.1%	*	2.1%	***
Vintage: Stage	17.1%	***	16.7%	***	10.4%	***	4.2%	***
Vintage: Maturity	2.4%	**	0.6%		3.3%	***	1.2%	**
Stage: Maturity	3.1%	**	5.3%	**	2.4%	**	2.1%	**
Treatment:Vintage:Stage	3.1%	*	2.4%		4.4%	***	3.0%	***
Treatment:Vintage: Maturity	0.3%		0.1%		0.94%	*	0.6%	
Treatment: Stage:Maturity	0.1%		0.2%		0.5%		1.6%	*
Vintage: Stage: Maturity	0.2%		0.1%		4.0%	***	4.5%	***
Residuals	13.1%		21.0%		8.0%		7.2%	

Significance is indicated as follow: * significant at 5%, ** significant at 1%, *** significant at 0.1% (ANOVA/T-test).

In order to study intra sample biodiversity (β-diversity), only the “SO_2_” and “Without SO_2_” treatments were taken into account, and 48H of maceration in the 2017 vintage was not taken into consideration as the ubiquity of *Saccharomyces cerevisiae* biased the data set for covariance analysis.

**FIGURE 5:**
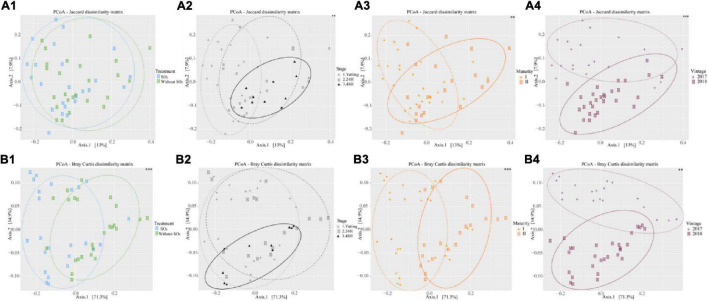
β – diversity by two matrix [Jaccard **(A)** and Bray Curtis **(B)**] in function of SO_2_ addition or not (1), Stage (Vatting, 24H and 48H of maceration, except 48 h maceration in 2017) (2), maturity (3) [technological (I) and advanced maturities (II)] and vintage (4) (2017 and 2018). Signif. codes for *p*-value (Manova): ^∗∗∗^0.001, ^∗∗^0.01.

Figure 5 presents the PCoA according to different parameters, with their significance obtained by MANOVA test (999 permutations). As for α-diversity, the use of the Jaccard matrix is qualitative, in that it considers only the nature of the OTUs, whereas the Bray-Curtis matrix takes their abundance into account. Concerning the Treatment parameter, the Jaccard matrix explained 13% of the variance on axis 1 and 7.9% on axis 2, whereas the Bray-Curtis matrix explained 71.3% on axis 1 and 14.9% on axis 2. The use of SO_2_ did not modify the relative composition of the OTUs (Figure 5A1), but did change their abundance (Figure 5B1). Conversely, Stage parameter affected the nature of the OTUs (Figure 5A2) but not their abundance (Figure 5B2). The fungal community structure, in terms of abundance, differed strongly between Maturity I and Maturity II (Figure 5B3) and between 2017 and 2018 (Figure 5B4), and to a lesser extent in terms of relative composition (Figure 5B4).

The percentage of variance explained by each parameter for β-diversity (Jaccard and Bray-Curtis) is presented in Table 2. Regarding the Jaccard matrix, each parameter impacted diversity significantly but at low percentages, and 54.8% of the variance remain unexplained by the parameters. For the Bray-Curtis matrix, Maturity, Treatment and Vintage impacted diversity significantly (22.7, 22.0, and 11.5% respectively) and 19.8% of the variance of the Bray-Curtis matrix was not explained by our parameters. Furthermore, the interaction of Vintage*Maturity was significant.

**TABLE 2:** Percentage of variance explained by Treatment (Without SO_2_ and SO_2_), Stage, Vintage, Maturity factors and the interactions for different β-biodiversity indices.

	Jaccard		Bray-Curtis	
Treatment	2.7%	*	22.0%	***
Vintage	6.1%	***	11.5%	***
Stage	5.9%	**	3.6%	
Maturity	4.3%	***	22.7%	***
Treatment:Vintage	1.7%		0.7%	
Treatment:Stage	4.3%		1.5%	
Treatment:Maturity	1.7%		2.1%	*
Vintage: Stage	2.2%		0.2%	
Vintage: Maturity	4.1%	***	8.0%	***
Stage: Maturity	4.3%		2.1%	
Treatment:Vintage:Stage	2.0%		0.5%	
Treatment:Vintage: Maturity	1.8%		1.9%	
Treatment: Stage:Maturity	2.9%		1.3%	
Vintage: Stage: Maturity	1.2%		2.1%	*
Residuals	54.8%		19.8%	

Significance is indicated as follow: * significant at 5%, ** significant at 1%, *** significant at 0.1% (Manova).

## Discussion

Nowadays, the use of sulfur dioxide is controversial due to consumers’ perceptions of its harmful effect and their demand for more “healthy food and beverages.” Moreover, with global warming, advanced maturity is a recurrent situation for harvests, and, as a consequence, fermentation management must take account of drastic changes in the matrix composition: high sugar content and pH, and elevated population levels of yeast and bacteria when grape berries become overripe (Martins et al., 2014). In this context, winemakers are being forced to redefine their winemaking processes, by reducing the use of SO_2_ and controlling the microbial community and alcoholic fermentation kinetics more effectively in order to preserve the sensorial quality of the wines. In the red winemaking process, the first addition of SO_2_ comes at the moment of vatting. Changes in the microbial yeast and fungal community during the first stages of the red winemaking process in the absence of SO_2_ may therefore have an impact on the alcoholic fermentation process and final wine quality.

In this work, the yeast and filamentous fungi microbial community was studied at different stages during prefermentary maceration (Vatting, 24H and 48H) using two complementary methodologies, Q-PCR to evaluate the population levels of target yeasts and metabarcoding to analyze microbial diversity. Different factors were considered: vinification practices (SO_2_, without SO_2_, Bioprotection), maturity level [technological (I), advanced (II)] and vintages (2017 and 2018).

The α-diversity was strongly impacted by the Stage, with a gradual decrease during the maceration process (Conacher et al., 2021). The yeast and filamentous fungi α-diversity have previously been shown to decrease during alcoholic fermentation, with the selection of dominant species (Nisiotou et al., 2007; David et al., 2014; Conacher et al., 2021). Our results showed that a decrease in α-diversity also occurred during prefermentary maceration; different factors, such as semi-anaerobiosis and low temperature (10°C), can explain the decrease in α-diversity, with the selection of yeast and filamentous fungi that are better suited to this environment.

### Little Impact of Vintage on α-Diversity, Unlike β-Diversity

It is well known that the microbial community is conditioned by the vintage, climate, and as a consequence, the sanitary status of the grape berries (Nisiotou et al., 2007; Bisson and Joseph, 2009; Bokulich et al., 2014; Steenwerth et al., 2021). The climatic conditions differed between the 2017 and 2018 vintages, with rainfall during September in 2017, while the weather was hot and dry during the harvest in 2018. This may explain the differences between the intra-sample diversity indices and their abundance. *Hanseniaspora* spp. dominated the fungal grape must community. Q-PCR and HTS analysis showed that *Hanseniaspora* spp. was strongly impacted by the vintage, with a higher population level and abundance in 2018 than in 2017. Previous studies showed a correlation between precipitation and humidity and population of *Hanseniaspora* spp. (Jara et al., 2016; Steenwerth et al., 2021). In the present work, precipitation during the harvest period was higher in 2017 than in 2018, and this climatic parameter therefore cannot explain our results. Another difference was highlighted between the two vintages: an indigenous population of *S. cerevisiae* colonized the grape juice at the end of the prefermentary maceration in 2017, whatever the treatment considered, but not in 2018. Since *S. cerevisiae* was not detected at vatting and after 24H of maceration, we can hypothesize that the origin of this population was the cellar, but not the grape berries.

### Yeast and Filamentous Fungi Diversity Were Impacted by the Maturity Level and Treatment

The nature and abundance of the OTUs differed between the two maturity levels. The impact of the maturity level on the abundance of the yeast and filamentous fungi community was high, with 22.7% of variance explained (*p*-value 0.001, Bray-Curtis). In the vineyard, modifications in the nutrient composition of grape berry exudates, particularly sugar exudation with advanced maturity, are likely to explain the changes in grape berry microbial community. Following this process, the grape must composition also differs both in sugar content and in pH between the two ripening stages, which can also have an impact on the diversity of microorganisms, including bacteria. For instance, some yeasts are able to persist and occupy the niche preferentially to others, resulting in a reduction in α-diversity in the grape must (Martins et al., 2012, 2014). Sanitary status should also be considered; in our study, the higher number of spores of *Botrytis cinerea* at technological maturity may explain why the native population levels of *Hanseniaspora* spp. in the initial must were higher at technological maturity than at advanced maturity (Rabosto et al., 2006; Liu et al., 2010).

The indigenous population level of *Metschnikowia pulcherrima* and *Torulaspora delbrueckii* was impacted negatively by SO_2_ addition at vatting. The first is one of the dominant species in the must, unlike the latter (Bokulich et al., 2014; David et al., 2014; Kecskeméti et al., 2016; Conacher et al., 2021). Non-*Saccharomyces* yeast is known to be sensitive to SO_2_, unlike *Saccharomyces cerevisiae* (Divol et al., 2012; Vicente et al., 2020). Surprisingly, the absence of sulfiting resulted in a decrease in yeast and fungal α-diversity during prefermentary maceration. Previous results have reported alteration of wine microbial diversity with sulfur dioxide treatment, but for white wine vinification, in which the antiseptic effect of SO_2_ is higher than in red wines (Bokulich et al., 2015; Morgan et al., 2019). In our study, the growth of species that are normally controlled by sulfur dioxide, in particular *Hanseniaspora* spp. (Albertin et al., 2014), could explain the decrease in α-diversity, by becoming the dominant species in the microbial community. Morgan et al. (2019) reported similar results on pinot gris, showing that the relative abundance of *Hanseniaspora* spp. was significantly higher in fermentations to which little or no SO_2_ had been added. The same result was observed by Grangeteau et al. (2017) in chardonnay, where the absence of SO_2_ led to a preponderance of indigenous yeasts such as *Hanseniaspora* spp., even preventing the occupation of the must by *Saccharomyces cerevisiae* and then leading to sluggish alcoholic fermentation. *Hanseniaspora* spp., particularly *H. uvarum*, can produce large amounts of ethyl acetate and acetic acid (Domizio et al., 2011; Jolly et al., 2014) and its presence at high levels is not desirable during prefermentary stages. However, used in mixed fermentation with *S. cerevisiae*, *H. uvarum* was shown to reduce volatile acidity and increase wine organoleptic quality (Tristezza et al., 2016; Capozzi et al., 2019). It is important to highlight that the population levels of the species were high (10^6^–10^7^ cells/mL) in our different experiments.

When comparing SO_2_ vs without SO_2_, there was little difference in β-diversity regarding the nature of the OTUs (Jaccard), but a significant impact of the treatment was highlighted when abundance was taken into consideration (22% of the variance explained, *p*-value 0.001). Our results, therefore, showed that SO_2_ altered the abundance of yeast and filamentous fungi, but not their nature.

### Bioprotection Yeasts Occupies the Niche With Higher Yeast Diversity Than in the Absence of SO_2_ Only

Until now, few studies have reported the effect of bioprotection on the grape juice yeast and filamentous fungi community as an alternative to sulfites (Simonin et al., 2018, 2020; Windholtz et al., 2021a,b). In the present study, a mix of two species, *Metschnikowia pulcherrima* and *Torulaspora delbrueckii*, was used as an alternative to sulfites. The two species had different behavior after inoculation throughout the prefermentary stages: *Torulaspora delbrueckii* grew to a high population level (>10^6^ cells/L), while the population of *Metschnikowia pulcherrima* remained stable. Low temperature during the maceration process (10°C), and a negative interaction with *Torulaspora delbrueckii*, through toxin killers for example (Villalba et al., 2016) or with the indigenous population of *Metschnikowia pulcherrima*, could explain the lower colonization of grape juice by *Metschnikowia pulcherrima.* The two species represented half of the relative abundance at technological maturity, but only 20–30% at advanced maturity, irrespective of the vintage considered. Lower abundance of bioprotection yeasts at advanced maturity could be explained by grape berries and grape juice microbial communities (yeast, filamentous fungi and bacteria) that differ from those at technological maturity. The ripening stage has been reported as impacting population level and diversity, with the highest abundance and diversity being found at the overripe stage (Martins et al., 2012, 2014). The greater occupation of the niche by some yeast species (hence the lower α-diversity at advanced maturity) through interaction phenomena could explain the lower abundance of bioprotective non-*Saccharomyces* at advanced maturity. As a consequence, it could be necessary to reconsider the doses applied at advanced maturity to manage bioprotection yeasts implantation and efficiency more effectively.

In a preliminary experiment on merlot, we reported on the occupation of the must by *Torulaspora delbrueckii* and *Metschnikowia pulcherrima* used as bioprotection, limiting the relative abundance of filamentous fungi, and in particular of *Aureobasidium* and *Botrytis* (Windholtz et al., 2021a). Our present data confirm that the use of these two species led to a decrease in the number of OTUs (Observed and Chao1), and especially in the numbers of OTUs of filamentous fungi that are systematically associated with a decline in grape must quality, such as *Aspergillus* spp. is likely to produce ochratoxin A in grape must, the main mycotoxin occurring in wine (Cabañes et al., 2002; Gil-Serna et al., 2018). On the other hand, biodiversity increased with the presence of bioprotection yeasts in the community, contrary to the use and the absence of SO_2_ (Shannon and InvSimpson). Bioprotection treatment has been shown to limit the colonization of *Saccharomyces* indigenous yeast at the end of the prefermentary stage when the population level was high (in 2017). This could facilitate the implantation of industrial Active Dry Yeast when added at the end of the cold maceration.

In previous studies, *Torulaspora delbrueckii* and *Metschnikowia pulcherrima* were recognized as species with antimicrobial potential (Oro et al., 2014; Ramírez et al., 2015; Villalba et al., 2016; Kántor et al., 2019). Johnson et al. (2020) showed a 44% reduction in acetic acid and 39% in population levels of *Hanseniaspora* spp. in cold maceration of grape juice with the use of these two species. In addition, they have shown that the impact of non-*Saccharomyces* yeast on the growth of *Hanseniaspora* spp. could depend on the strains present in the medium, with a high intra-specific genetic diversity being reported for this species (Albertin et al., 2016). In our experimental conditions, the use of bioprotection had no impact on *Hanseniaspora* spp. population levels but did have a negative effect on its abundance. One hypothesis to explain this result is the very high initial population level of indigenous *Hanseniaspora* spp. (>10^6^ cells/ml) compared to our previous study in the 2018 vintage with merlot grapes from the Bordeaux area (10^4^ cells/mL) (Windholtz et al., 2021a).

## Conclusion

The impact of various parameters on the yeast and filamentous fungi microbial community during the prefermentary stages of the winemaking process without sulfites was highlighted by this study. Intra-sample diversity of the yeast and filamentous fungi community remained stable from one vintage to another, but its intrinsic composition changed. A decrease in diversity could be observed during the prefermentary stage, as the species present on the grape berry finally gave way to those better suited to the grape must environment. Finally, the absence of sulfiting led to an occupation of the population niche by some dominant species, but which were not particularly desirable (such as *Hanseniaspora* spp.), leading to an unexpected reduction in diversity compared to the presence of SO_2_. On the other hand, the OTUs were similar in both treatments, with and without sulfites, and only their abundance varied. Niche colonization by bioprotection yeasts was effective in all of the trials, although *Torulaspora delbrueckii* showed growth, unlike *Metschnikowia pulcherrima*, which remained stable. Moreover, their relative abundance was lower at advanced maturity. The use of bioprotective non-*Saccharomyces* yeast limited the abundance of filamentous fungi that are systematically associated with a decline in grape must quality. Study of the bacterial community should make it possible to complete these results. It would also be interesting to further include grapes from others varieties and with different sanitary status in the same experiment for additional vintages to expand our knowledge of the prefermentary microbial community in a context of vinification without sulfites.

## Data Availability Statement

The original contributions presented in the study are included in the article/Supplementary Material, further inquiries can be directed to the corresponding author. The datasets generated for this study can be found in NCBI (ID PRJNA772556).

## Author Contributions

SW: conceptualization, methodology, investigation, data curation, formal analysis, visualization, and writing – original draft. EV: resources and investigation. LF: investigation. CT: conceptualization and supervision. IM-P: conceptualization, project administration, supervision, and writing – review and editing. All authors contributed to the article and approved the submitted version.

## Conflict of Interest

The authors declare that the research was conducted in the absence of any commercial or financial relationships that could be construed as a potential conflict of interest.

## Publisher’s Note

All claims expressed in this article are solely those of the authors and do not necessarily represent those of their affiliated organizations, or those of the publisher, the editors and the reviewers. Any product that may be evaluated in this article, or claim that may be made by its manufacturer, is not guaranteed or endorsed by the publisher.
