# Supplementary material for: Yeast and Filamentous Fungi Microbial Communities in Organic Red Grape Juice: Effect of Vintage, Maturity Stage, SO2, and Bioprotection
Source: Front Microbiol. 2021 Dec 24;12:748416. doi: 10.3389/fmicb.2021.748416 (PMC8740202; doi:10.3389/fmicb.2021.748416)
Supplement: Supplementary file 1 [file Data_Sheet_1.PDF]

Fig.S.1: Population dynamics of *Metschnikowia pulcherrima* , *Torulaspora delbrueckii* and *Hanseniaspora* sp. during prefermentary stages (Vatting, 24H and 48H of maceration) in two vintages (2017 and 2018) and two maturities (technological (A) and advanced maturities (B)). Bioprotection (BP), SO_2 and Without SO_2 treatments in early stages of winemaking

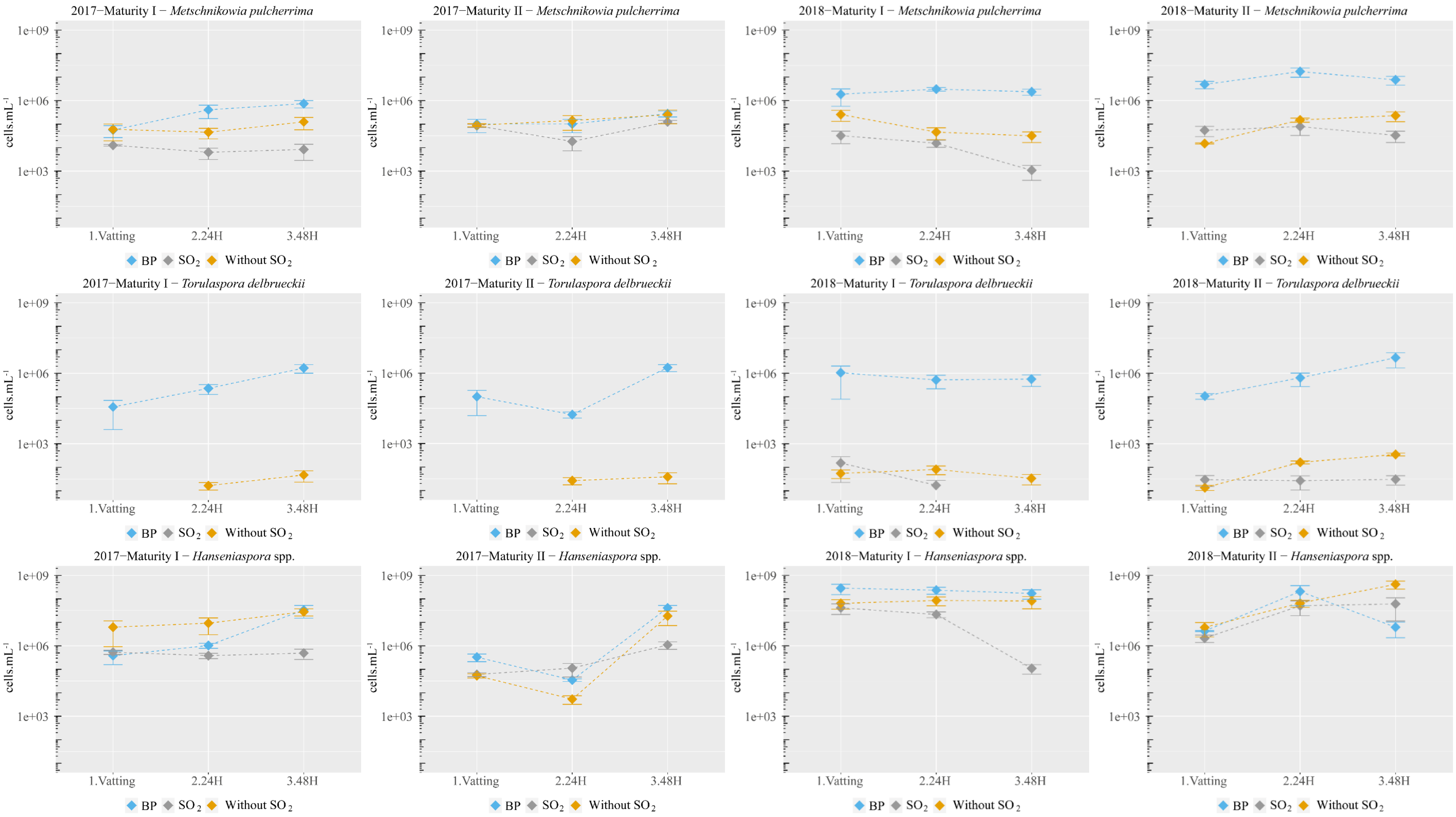

Fig.S.2: Relative abundances (%) of major genus of *Ascomycota* phylum, filamentous fungi, for all sample during prefermentary stages (Vatting, 24H and 48H of maceration) in two vintages (2017 and 2018) and two maturity levels (technological (I) and advanced maturities (II)). Bioprotection (BP), SO_2 and Without SO_2 treatments in early stages of winemaking. Results of each sample is the mean of biological replicats (n=3 technological maturity, n=2 advanced maturity)

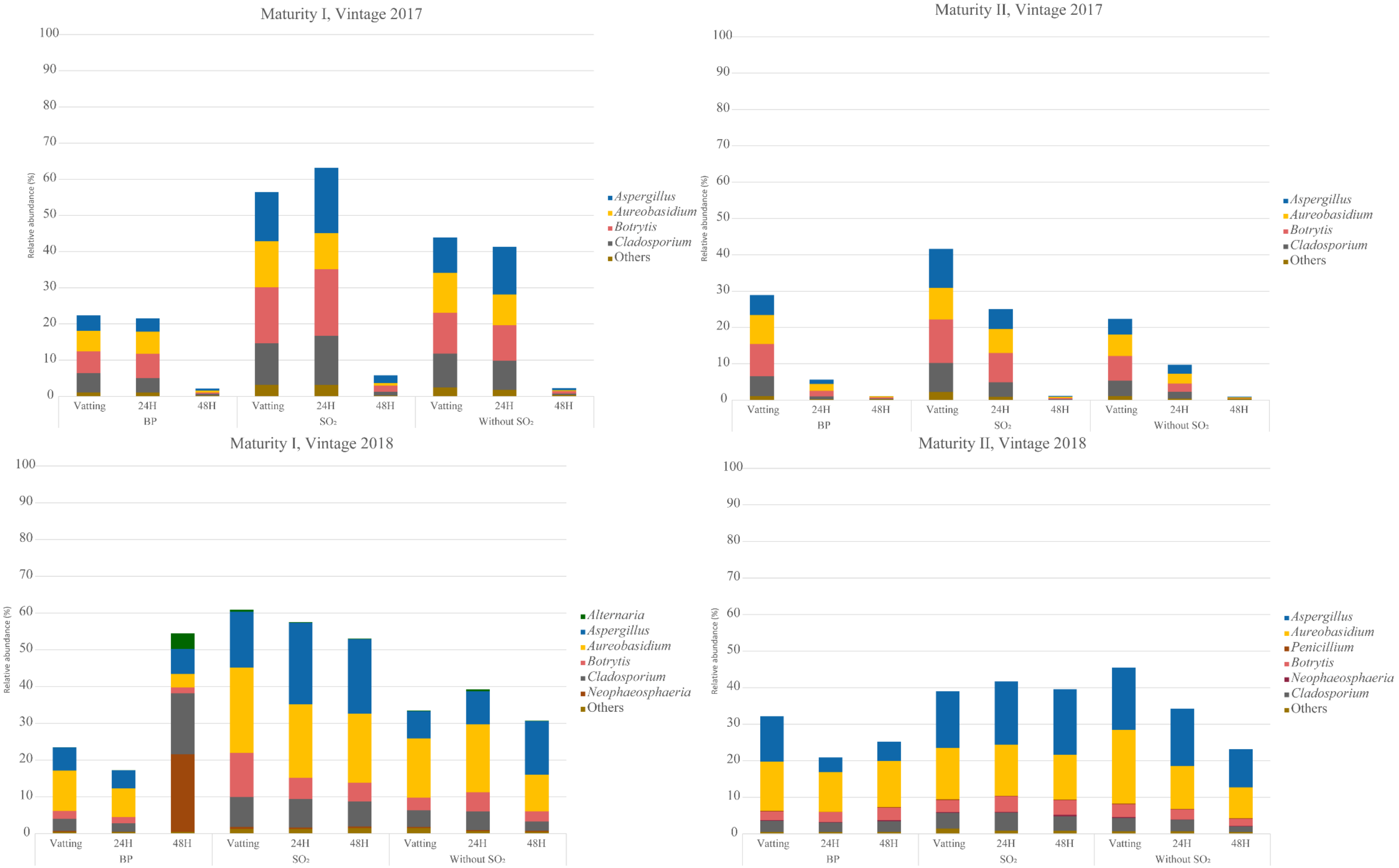

Table S.1: Primers used to quantify population levels of microorganisms

Species	Primers	References
Metschnikowia pulcherrima	MP2-F AGACACTTAACTGGGCCAGC<br>MP2-R GGGGTGGTGTGGAAGTAAGG	(García *et al.*, 2017)
Torulaspora delbrueckii	TD-F CAAAGTCATCCAAGCCAGC<br>TD-R TTCTCAAACAATCATGTTTGGTAG	(Zott *et al.*, 2010)
Hanseniaspora spp.	Hauf 2L — CCCTTTGCCTAAGGTACG<br>Hauf 2R — CGCTGTTCTCGCTGTGATG	(Zott *et al.*, 2010)

Table S.2: Grape must composition for two vintages (2017 and 2018) and maturity levels (technological (I) and advanced maturity (II)). Bioprotection (BP), SO_2 and Without SO_2 treatments in early stages of winemaking. (±: standard deviation of three and two biological replicates, for maturity I and II, respectively)

	Harvest date	Treatments	Potential ethanol (% v/v)	pH	Sugars g/L	spores of *B. cinerea*/mL
2017-I	11/10/2017	SO_2	13.1 ± 0	3.46 ± 0.006	222 ± 0	1.07×10^7 ± 0.15
		Without SO_2	13.0 ± 0	3.44 ± 0.006	221 ± 0	5.90×10^6 ± 2.34
		Bioprotection	12.8 ± 0	3.47 ± 0.012	217 ± 0.6	1.75×10^7 ± 0.07
2017-II	18/10/2017	SO_2	13.4 ± 0	3.58 ± 0.000	227 ± 4.2	5.20×10^6 ± 1.98
		Without SO_2	13.8 ± 0	3.57 ± 0.000	234 ± 4.2	8.15×10^6 ± 1.98
		Bioprotection	13.3 ± 0	3.55 ± 0.000	226 ± 0.7	9.50×10^6 ± 3.19
2018-I	18/09/2018	SO_2	13.4 ± 0	3.57 ± 0.010	223 ± 2.5	1.36×10^7 ± 0.41
		Without SO_2	13.3 ± 0	3.57 ± 0.006	221 ± 2.1	6.67×10^6 ± 1.80
		Bioprotection	13.3 ± 0	3.57 ± 0.006	220 ± 1.6	1.90×10^7 ± 0.57
2018-II	25/09/2018	SO_2	13.9 ± 0	3.61 ± 0.000	233 ± 1.4	5.35×10^6 ± 4.10
		Without SO_2	13.8 ± 0	3.61 ± 0.000	231 ± 1.4	1.60×10^6 ± 1.80
		Bioprotection	14.0 ± 0	3.60 ± 0.000	235 ± 0.7	5.95×10^6 ± 5.66

Table S.3: Analysis of wine after alcoholic fermentation, for two vintages (2017 and 2018) and two maturity levels (technological (I) and advanced maturity (II)). Bioprotection (BP), SO_2 and Without SO_2 treatments in early stages of winemaking. Total SO_2 analysis was performed after malolactic fermentation. (±: standard deviation of three and two biological replicates, for maturity I and II, respectively)

	Treatments	Ethanol (v/v)	Volatile acidity (acetic acid g/L)	pH	Total acidity (tartaric acid g/L)	Total SO_2 (mg/L)
2017-I	SO_2	13.5 ± 0.05	0.12 ± 0.01	3.75	5.9 ± 0.09	19 ± 1.4
	Without SO_2	13.5 ± 0.00	0.11 ± 0.01	3.74	6.0 ± 0.00	< 10
	Bioprotection	13.4 ± 0.04	0.12 ± 0.01	3.75	6.0 ± 0.15	< 10
2017-II	SO_2	14.2 ± 0.13	0.12 ± 0.00	3.83	5.7 ± 0.11	26 ± 0.0
	Without SO_2	14.1 ± 0.14	0.11 ± 0.01	3.82	5.9 ± 0.11	< 10
	Bioprotection	14.1 ± 0.15	0.12 ± 0.00	3.82	5.9 ± 0.11	< 10
2018-I	SO_2	13.5 ± 0.07	0.14 ± 0.03	3.80	5.8 ± 0.11	30.5 ± 2.1
	Without SO_2	13.5 ± 0.05	0.13 ± 0.02	3.78	5.9 ± 0.09	< 10
	Bioprotection	13.5 ± 0.02	0.11 ± 0.01	3.79	5.9 ± 0.09	< 10
2018-II	SO_2	14.3 ± 0.11	0.12 ± 0.02	3.82	5.7 ± 0.11	28.5 ± 0.7
	Without SO_2	14.3 ± 0.08	0.09 ± 0.01	3.83	5.8 ± 0.00	< 10
	Bioprotection	14.3 ± 0.09	0.09 ± 0.00	3.83	5.8 ± 0.00	< 10

Table S.4: Percentage of variance explained by Treatment (Without SO_2, SO_2 and Bioprotection), Stage, Vintage, Maturity parameters and their interactions on population levels of *Hanseniaspora* spp (Hu), *Metschnikowia pulcherrima* (Mp) and *Torulaspora delbrueckii* (Td)

	Hu		Mp		Td	
Treatment	8.6%	***	29.7%	***	77.4%	***
Vintage	25.8%	***	6.0%	***	1.1%	***
Stage	1.7%	*	0.2%		0.9%	**
Maturity	3.7%	***	1.4%	*	0.2%	
Treatment :Vintage	0.4%		6.3%	***	0.1%	
Treatment :Stage	6.8%	***	5.0%	**	3.9%	***
Treatment : Maturity	0.2%		1.9%	*	0.1%	
Vintage: Stage	10.5%	***	2.4%	*	0.8%	
Vintage : Maturity	0.20%		0.9%		0.1%	
Stage : Maturity	4.4%	***	0.8%		0.6%	
Treatment :Vintage :Stage	0.3%		0.8%		0.6%	
Treatment :Vintage : Maturity	2.2%	**	0.4%		0.02%	
Treatment : Stage :Maturity	2.1%	*	3.3%		0.5%	
Vintage : Stage : Maturity	1.4%		1.2%		0.9%	**
Residuals	31.9%		39.0%		13.24%	
